# Isolated AL Amyloidosis of the Colon: A Rare Presentation

**DOI:** 10.14309/crj.0000000000001277

**Published:** 2024-02-07

**Authors:** Alexander Garcia, Mahir Qureshi, Ishita Dhawa, William Rafferty, Tulin Budak-Alpdogan, Samuel Giordano

**Affiliations:** 1 Department of Medicine, Cooper University Hospital, Camden, NJ; 2 Department of Gastroenterology, Cooper University Hospital, Camden, NJ; 3 Department of Pathology, Cooper University Hospital, Camden, NJ; 4 Department of Hematology/Oncology, Cooper University Hospital, Camden, NJ

**Keywords:** gastrointestinal tract, amyloidosis, colon, dysmotility

## Abstract

Amyloidosis is a group of rare deposition diseases marked by the accumulation of abnormal fibrillar proteins in the extracellular space of various tissues. In both AL and AA amyloidosis, the most common variants, isolated involvement to any one organ is uncommon and involvement to the colon alone is especially rare. We present the case of a patient who was initially found to have AL amyloidosis on prior screening colonoscopy that was reconfirmed several years with repeat evaluation for chronic constipation. This disease process is often insidious and can be overlooked by providers given the lack of overwhelming symptoms.

## INTRODUCTION

Amyloidosis, a heterogeneous group of diseases, is characterized by the deposition of abnormally folded proteins in the extracellular space.^1–3^ While 6 types exist, the most commonly encountered types include primary amyloid, also known was AL amyloid (light chain disease), and secondary/reactive amyloid (AA protein disease). AL amyloidosis is estimated to affect 6.13 per million person-years, and AA amyloidosis is estimated to affect 1.21 million person-years.^4^ In the former disease, monoclonal proliferation of plasma cells leads to the overproduction and deposition of immunoglobulin light chains (kappa and lambda) in peripheral tissues, leading to organ dysfunction and an array of clinical manifestations. As systemic diseases, both AL and AA amyloidosis often involved the gastrointestinal (GI) tract; however, isolated involvement of the GI alone is quite rare.^5^ We present the case of a patient with chronic constipation who went on to be diagnosed with isolated AL amyloidosis of the colon.

## CASE REPORT

A 61-year-old man with a history of hypertension and benign colonic polyps presented to our gastroenterology practice to re-establish care and for assessment of chronic constipation. Several years earlier, the patient reported frequent straining with difficulty passing bowel movements. At this time, on screening colonoscopy, the ascending colon had nodular erythema. Pathological analysis of the involved areas revealed colonic mucosa with nodular amyloid. Positivity for birefringence, Congo red, and crystal violet confirmed a diagnosis of amyloid. Further outpatient workup with hematology/oncology during this time went on to reveal a negative serum protein electrophoresis without an M-spike and only slightly elevated free kappa light chains (20 mg/L), favoring isolated local disease instead of systemic involvement. Unfortunately, the patient was subsequently lost to follow-up and would not re-present until several years later.

**Figure 1.**
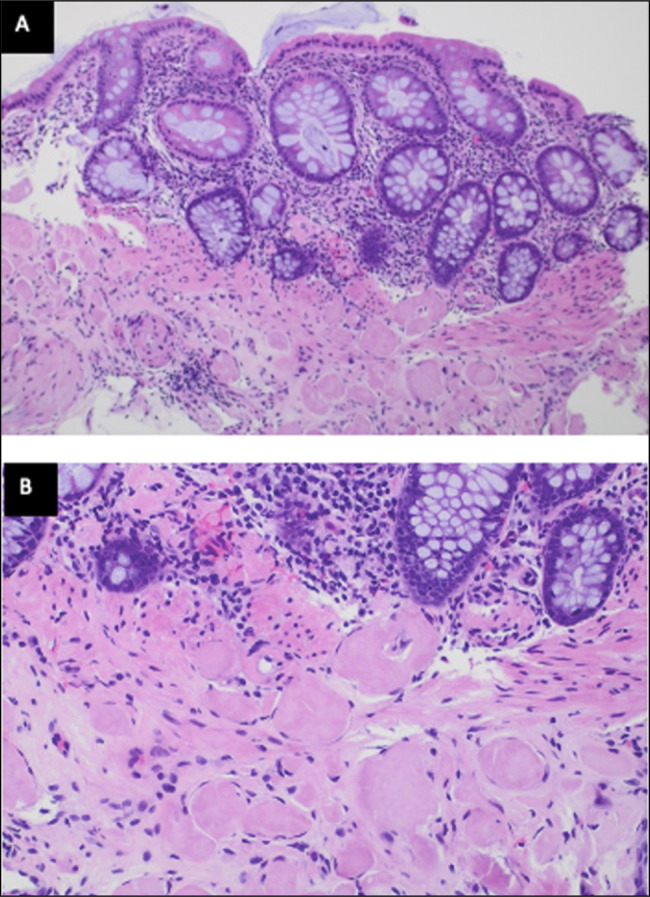
Colonic mucosa showing nodular amyloid deposits in the muscularis mucosa in (A) 100× and (B) 200× magnification, respectively.

**Figure 2.**
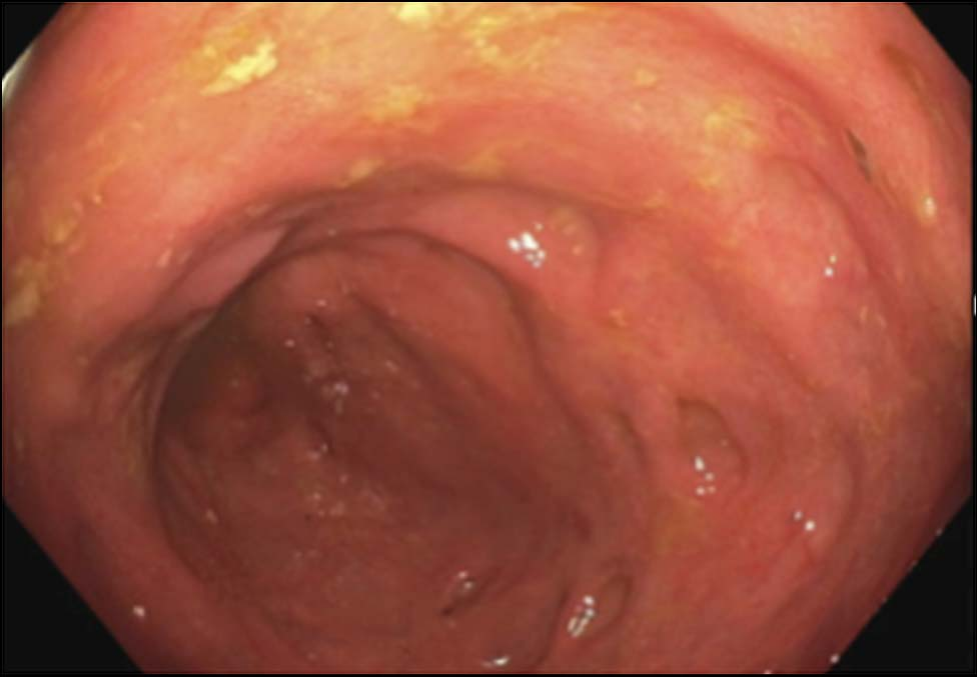
Erythematous mucosa in the ascending colon.

After re-establishing care, the patient underwent repeat colonoscopy for assessment of constipation with associated abdominal bloating and nausea. On repeat colonoscopy, he was found to have a 5 mm polyp in the transverse colon. The remaining colon was otherwise normal in appearance without any irregularities. Cold forceps polypectomy was completed on the visualized transverse polyp, and random colonic biopsies were obtained throughout the colon given the history of isolated amyloid. Pathology from the removed polyp went on to reveal tubular adenoma with amyloid deposits involving the lamina propria. The random tissue samples retrieved from the right colon, transverse colon, and left colon were all positive for amyloid deposits with characteristic findings of a positive Congo red stain and polarization (Figure 1). Gross appearance showed erythematous mucosa including in the ascending colon (Figure 2). Mass spectrometry demonstrated a diagnosis most consistent with AL amyloidosis, lambda type. Serum kappa light chain was elevated to 26.1 from 20.0 4 years earlier (range 3.3–19.4 mg/L), and serum lambda light chain was normal. Bone marrow biopsy was negative for myeloma workup. Repeat serum protein electrophoresis did not reveal an elevated M-spike, which favors the diagnosis of isolated AL amyloidosis of the colon. Bone survey was negative for lytic or blastic lesions. At the present time, further oncological workup is pending. However, the patient has barriers for follow-up and further workup.

## DISCUSSION

Amyloidosis is a rare disease process highlighted by the accumulation of misfolded proteins in the extracellular space. In the 2 most common forms of this disease, AL and AA amyloidosis, systemic involvement often includes the GI tract, with patients exhibiting a large array of GI symptoms including diarrhea, abdominal pain, malabsorption, esophageal reflux, and gastrointestinal bleeding including life-threatening hemorrhage.^4^ Moreover, if the small bowel is affected, the duodenum tends to be the most involved region.^4,6^ AL amyloidosis typically requires systemic treatment such as chemotherapy and/or hematopoietic stem cell transplant.^7^ As in our patient, AL amyloidosis commonly presents amyloid deposits in the form of a mass in the submucosal layer and muscularis propria layer of the GI tract.

Isolated colonic involvement by amyloid, as in our case, however, is quite rare. Localized colonic involvement can present with symptoms of rectal bleeding, abdominal pain, anemia, intestinal obstruction, or weight loss.^8–14^ There is also a reported case of a patient with isolated colonic amyloidosis and positive abdominal fat pad biopsy who was asymptomatic at the time of presentation but did have a positive fecal occult blood test.^3^ Interestingly, our patient presented with a chief concern initially with frequent straining with difficulty passing bowel movements and later symptoms of constipation, which is an uncommon symptom of GI amyloidosis.^1^ The patient did not have any other symptoms, medications, or medical history that could reasonably explain these findings. Another interesting component is that the biopsy showed AL amyloidosis throughout the colon. Amyloid deposits tend to be localized within specific regions of the colon, with the colorectum and transverse colon being the 2 most common areas of involvement.^5^ By contrast, our patient had pan-colon involvement, which has not been reported in the literature before to our knowledge.

Interesting to note, a prior study highlighted the prognostic factors for patients with localized AL amyloidosis in various organ sites. Most patients presented asymptomatically. Patients also responded to treatment, but 40% showed local progression. The 5-year localized AL amyloidosis progression-free survival rate was similar for patients with varying organ sites. In addition, at 5 years from treatment, 41% of responders of treatment and 71% with stable disease did demonstrate evidence of local progression. Different treatment regimens including chemotherapy, steroids, and/or surgery did not demonstrate any significant differences in outcomes.^15^ However, in our patient, initial presentation involved episodes of constipation, and it is difficult to ascertain whether the amyloidosis and constipation are related. In addition, the patient has not initiated treatment yet.

Given the current reported case reports of amyloid deposit findings in the gastrointestinal tract, it is imperative that a broad differential is formed when patients present with minimal or nonspecific symptoms to ensure the correct diagnosis is made. Examination through endoscopy or colonoscopy with biopsies of targeted areas is warranted if amyloidosis is suspected. A full workup is indicated to determine whether the disease process is localized or systemic. The type of amyloidosis should also be confirmed to initiate proper treatment if indicated.

## DISCLOSURES

Author contributions: A. Garcia, M. Qureshi, and I. Dhawan: created and reviewed the manuscript. A. Allen: reviewed pathology report, reviewed manuscript. T. Budak-Alpdogan and S. Giordano: reviewed manuscript. S. Giordano is the article guarantor.

Financial disclosure: None to report.

Informed consent was obtained for this case report.
